# Development of a loop-mediated isothermal amplification (LAMP)-based electrochemical test for rapid detection of SARS-CoV-2

**DOI:** 10.1016/j.isci.2023.107570

**Published:** 2023-08-09

**Authors:** Khushboo Borah Slater, Muhammad Ahmad, Aurore Poirier, Ash Stott, Bianca Sica Siedler, Matthew Brownsword, Jai Mehat, Joanna Urbaniec, Nicolas Locker, Yunlong Zhao, Roberto La Ragione, S. Ravi P. Silva, Johnjoe McFadden

**Affiliations:** 1 School of Biosciences, Faculty of Health and Medical Sciences, University of Surrey, Guildford GU2 7XH, UK; 2 Advanced Technology Institute, University of Surrey, Guildford GU2 7XH, UK; 3 School of Veterinary Medicine, Faculty of Health and Medical Sciences, University of Surrey, Guildford GU2 7AL, UK

**Keywords:** Diagnostic technique in health technology, Devices

## Abstract

Rapid, reliable, sensitive, portable, and accurate diagnostics are required to control disease outbreaks such as COVID-19 that pose an immense burden on human health and the global economy. Here we developed a loop-mediated isothermal amplification (LAMP)-based electrochemical test for the detection of SARS-CoV-2 that causes COVID-19. The test is based on the oxidation-reduction reaction between pyrophosphates (generated from positive LAMP reaction) and molybdate that is detected by cyclic voltammetry using inexpensive and disposable carbon screen printed electrodes. Our test showed higher sensitivity (detecting as low as 5.29 RNA copies/μL) compared to the conventional fluorescent reverse transcriptase (RT)-LAMP. We validated our tests using human serum and saliva spiked with SARS-CoV-2 RNA and clinical (saliva and nasal-pharyngeal) swab samples demonstrating 100% specificity and 93.33% sensitivity. Our assay provides a rapid, specific, and sensitive test with an electrochemical readout in less than 45 min that could be adapted for point-of-care settings.

## Highlights

• Rapid detection of SARS-CoV-2 using a LAMP-based electrochemical test
• The test is sensitive in detecting as low as 5.29 RNA copies/μL
• Validation of the electrochemical test in human serum and saliva samples
• The test was 100% specific and 93.33% sensitive in detecting SARS-CoV-2

## Graphical abstract

**Figure:**
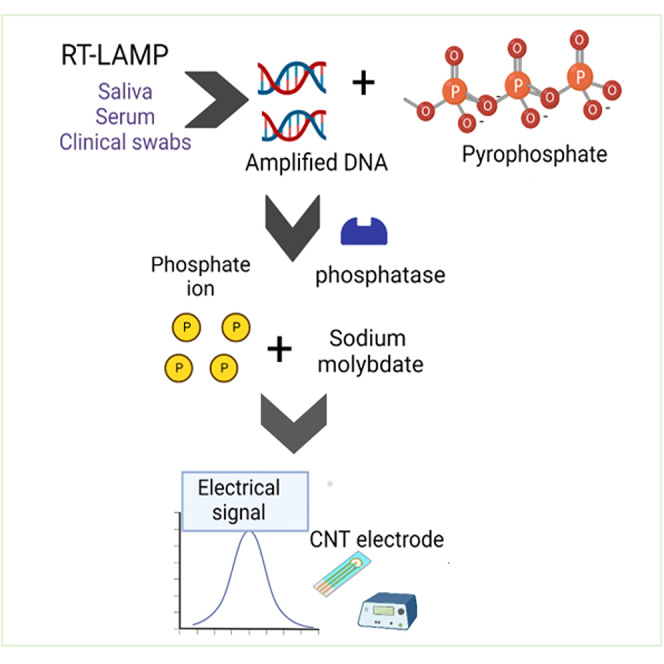

## Introduction

The coronavirus SARS-CoV-2 was declared a global COVID-19 pandemic on March 2020 by the World Health Organization (WHO). Nearly six million deaths have since been recorded, with around two million new cases registered every 24 h globally.^1^ SARS-CoV-2 is a virus belonging to the family of coronaviruses with positive-sense RNA and a genome size of ∼29.9 kb and 13 to15 open reading frames.^2^^,^^3^ The virus comprises four main structural proteins: spike (S), membrane (M), envelope (E), and nucleocapsid (N). SARS-CoV-2 shares more than 80% sequence identity with SARS-CoV-1 (that caused a previous outbreak in China in 2003), and around 50% with Middle East respiratory syndrome coronavirus (MERS-CoV) (a virus transmission identified from camels to humans in 2013) revealing a common pathogenicity between the three coronaviruses.^2^^,^^3^^,^^4^ Coronaviruses have been proposed to be zoonotic in origin and transmission; however, in the case of SARS-CoV-2, a specific natural animal reservoir or origin is yet to be confirmed.^4^^,^^5^^,^^6^ COVID-19 mainly affects the respiratory system causing cough, fever, and difficulty in breathing, cardiac injury, respiratory failure, and multi-organ dysfunction in severe cases.^2^ The virus is highly transmissible and spreads through air droplets and by contact with infected individuals.^2^ With the likelihood of this virus continuously evolving and coexisting with humans for a long time, there is a need for continuous efforts in developing efficient diagnostics to control and manage this disease, and to prevent future outbreaks.^7^ This will depend primarily on our ability to detect the virus of interest with a high level of sensitivity using inexpensive technologies that can be rapidly deployed.

Rapid and accurate identification of microbial agents such as virus or bacteria that cause an infection is key for the treatment, care, management, and control of the spread of COVID-19 and any other infectious disease. This also includes infection in animals, such as in the case of the swine flu or avian influenza. Misdiagnosis or inaccuracies in diagnosis leads to adverse clinical outcomes and even mortality, such as reported in cases of COVID-19, tuberculosis, respiratory tract infections, and sepsis diagnosis.^8^^,^^9^^,^^10^ Sensitive and specific diagnostic tests are therefore crucial to provide accurate identification of the cause of the disease, and appropriate timely treatment and control measures.^11^ Advances in molecular techniques have revolutionized diagnostics in development of fast, reliable, efficient, and cost-effective techniques. Molecular diagnostics such as polymerase chain reaction (PCR), loop-mediated isothermal amplification (LAMP) and antigen tests enabled rapid identification of COVID-19 pandemic, even in low-resource public health settings across the world.^12^ Okeke et al., 2021 reviewed a rapid expansion of public health care settings (4 to 72) in Nigeria equipped to undertake molecular diagnostic tests for COVID-19 between the period Jan 2020-2021.^12^ An ideal diagnostic test for resource-limited settings is one that satisfies the ASSURED criteria (affordable, sensitive, specific, rapid, user-friendly, equipment-free, and deliverable to end users)^13^ and one that does not rely on cold chain-dependent transport of specimen.^14^ Challenges remain to develop such tests that satisfy the ASSURED criteria^13^ for COVID-19 and new variants and other zoonotic pathogens.

Reverse transcription polymerase chain reaction (RT-PCR) is the standard diagnostic technique for detecting COVID-19.^15^^,^^16^^,^^17^ The wider application of this technique in low resource settings during a pandemic is a problem since it requires RNA extraction from samples, reagents, sophisticated laboratory equipment, and trained personnel to carry out the assays.^15^^,^^18^^,^^19^ Moreover, this assay is time consuming and is prone to a higher risk of inhibitions by reaction components and contaminations.^15^ LAMP is one of the most widely used isothermal amplification technique; an alternative to qPCR and is a faster technique involving a single step isothermal amplification method for nucleic acids that can amplify a few copies of target to 10^9^ copies within 30 min.^20^^,^^21^ LAMP uses four to six primers that bind six to eight distinct regions of the target DNA, making it highly specific for recognition of the target sequence and a strand displacing DNA polymerase that initiates synthesis.^20^ LAMP reaction can be conducted at a single temperature (isothermal amplification) using simple instruments such as a water bath or a heat block, which makes it an easily adaptable technique predominantly in the laboratory or diagnostic settings.^15^ There are multiple modes of detecting LAMP amplified products such as fluorometry, colorimetry and turbidity. Moreover, LAMP is superior to traditional PCR in rapid point-of-care (POC) diagnostic settings as it is less sensitive to PCR inhibitors present in clinical samples such as in saliva, blood, urine and produces large number of amplified products and its by-product magnesium pyrophosphate that are easily detectable with colorimetry and turbidity.^15^^,^^16^^,^^20^^,^^22^^,^^23^^,^^24^

Multiple studies have optimized LAMP technology for the detection of SARS-CoV-2 as POC diagnostics in low resource settings.^12^^,^^25^^,^^26^^,^^27^ Gartner et al., 2022 optimized LAMP assay to enable detection of SARS-CoV-2 in inactivated and non-extracted nasopharyngeal and throat swab samples collected in Malawi providing inexpensive diagnostics in resource-limited settings.^27^ A different study used a combination of N and S, two target genes for fluorometric RT-LAMP detection of clinical SARS-CoV-2 isolate.^18^ However, carryover or cross contaminations are problems with LAMP assays due to aerosols formed with a large number of amplified products in a positive reaction, and if reaction tubes are not handled carefully these can contaminate the surrounding area leading to false positives.^15^ SARS-CoV-2 LAMP technique has also been coupled with CRISPR and nanopore sequencing to improve detection and accuracy.^28^^,^^29^ A colorimetric LAMP-based molecular diagnostic device that used integrated imaging and artificial intelligence-based image processing to detect SARS-CoV-2 has also been developed. However, this test is based on the pH change from a positive LAMP reaction and any changes in pH during extraction process may lead to false positives.^30^ In this study we developed an electrochemical-rapid diagnostic test (RDT) which detects an electrical signal generated from oxidation-reduction (redox) reactions of pyrophosphates (produced in a positive RT-LAMP reaction) with sodium molybdate. We optimized our electrochemical-RDT to work with human fluids such as serum and saliva that contains inherent phosphate contaminants. Our assay provides rapid and sensitive detection of SARS-CoV-2 in clinical samples and a platform for development of new POC diagnostics.

## Results

### Development of SARS-CoV-2 fluorescent RT-LAMP

**Figure 1:**
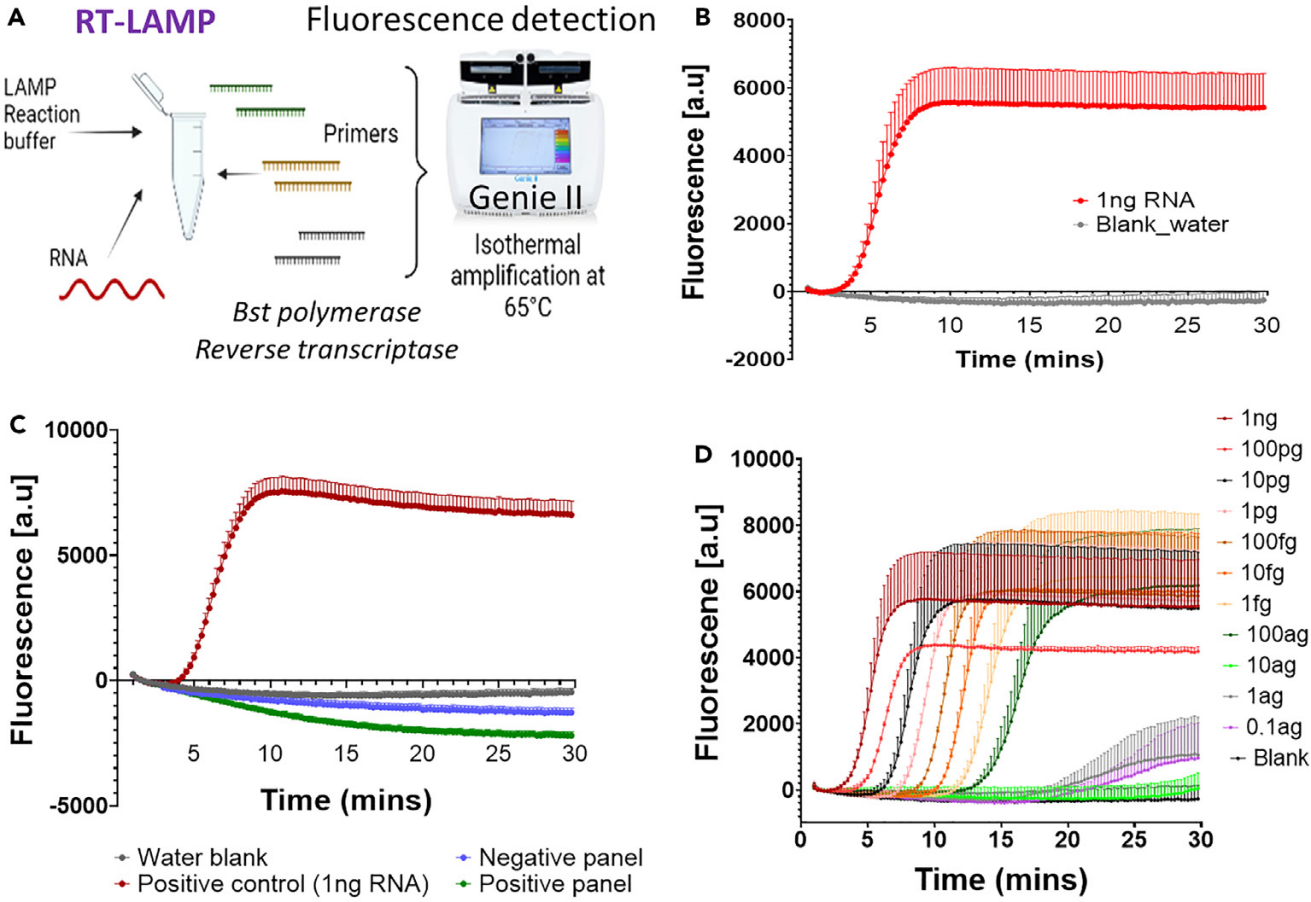
Development of SARS-CoV-2 fluorescent LAMP (A) Schematic of fluorescent RT-LAMP detection. Six LAMP primers comprising outer F3/B3, looping inner FIP/BIP and loop primers LF/LB were designed to target M gene of SARS-CoV-2. Primers, *Bst* polymerase, RT reverse transcriptase and isothermal reaction buffer were added together to set up the LAMP reaction. RNA was added as the template to the LAMP reaction and the amplification was carried out isothermally at 65°C for 30 min in a Genie II instrument as shown in the scheme. Real time fluorescence was monitored over 30 min. (B) SARS-CoV-2 M gene RNA detection with fluorescent LAMP. Values are mean ± Standard error of the mean (SEM) for both positive (1ng RNA templates) and blank water (n = 4). (C) Cross specificity test for M gene LAMP. Positive panel includes DNA and RNA from 21 respiratory pathogens. Negative panel includes inactivated DNA and RNA from the same 21 respiratory pathogens. Values are mean ± SEM (n = 4). (D) LOD analysis for M gene fluorescent LAMP. Values are mean ± SEM (n = 4). Figure reference Biorender.com; OptiGene.

The membrane protein (M) gene of SARS-CoV-2 was targeted for designing a fluorescent LAMP based assay. The M protein is the most abundant structural protein essential for virus assembly and morphogenesis and is an attractive target for RT-LAMP assays.^24^^,^^31^^,^^32^ Six primers: forward and backward loop primers (LF, LB), forward and backward outer and internal primers (F3, B3, FIP, BIP) were designed to amplify an M gene fragment of 669bp (See Table 1 for primers, Data S1 for M gene sequence). A defined LAMP reaction buffer (see STAR methods) was developed to obtain optimal DNA amplification and to minimize any cross reactivities from the buffer components (such as potassium chloride (KCL) and dioxyribonucleotides dNTPs). We used synthetic RNA of M gene to develop the LAMP test (see STAR methods). The fluorescence detection for M gene targeting LAMP assay is shown in Figures 1A, 1B, and S1A–S1C. Amplification was achieved within 15 min confirming the specificity of the primers. We validated the analytical specificity of M gene targeting LAMP assay by checking the cross specificity of the primers using multiple DNA and RNA as template from 21 respiratory pathogens and avian IBV coronavirus (Figures 1C and S1D; see Table S3 for the list of respiratory pathogens tested). The cross-specificity test demonstrated 100% specificity of the primers for SARS-CoV-2 M gene. We tested the limit of detection (LOD) for the fluorescence-based assay by diluting synthetic RNA from 1ng to 10^−10^-fold (0.1 ag) in nuclease free water. The LOD of fluorescent assay was recorded to be 10^− to7^-fold dilution of 1ng starting template (100 ag) which accounts for 52.95 copies of ssRNA per reaction or 5.29 X 10^4^ copies/mL (Figure 1D). We used this working SARS-CoV-2 fluorescent LAMP assay as a reference test for the electrochemical assay which is the focus of this study.

**Table 1:** LAMP primers for amplifying M gene of SARS-CoV-2

Primer	Primer sequences 5′ to 3′
F3	TTGCTATCGCAATGGCTT
B3	TGATGTCACAGCGTCCTA
FIP(F1c+F2)	GGAGTGGCACGTTGAGAAGAAT GCTTCTTTCAGACTGTTTGC
BIP(B1c+B2)	GGCACTATTCTGACCAGACCGGATGTC CACGAAGGATCAC
LoopF	GGATTGAATGACCACATGGAAC
LoopB	AGTGAACTCGTAATCGGAGC
F2	GCTTCTTTCAGACTGTTTGC
F1c	GGAGTGGCACGTTGAGAAGAAT
B2	GATGTCCACGAAGGATCAC
B1c	GGCACTATTCTGACCAGACCG
Product	Sequence Length:669

### Development of SARS-CoV-2 electrochemical-RDT

**Figure 2:**
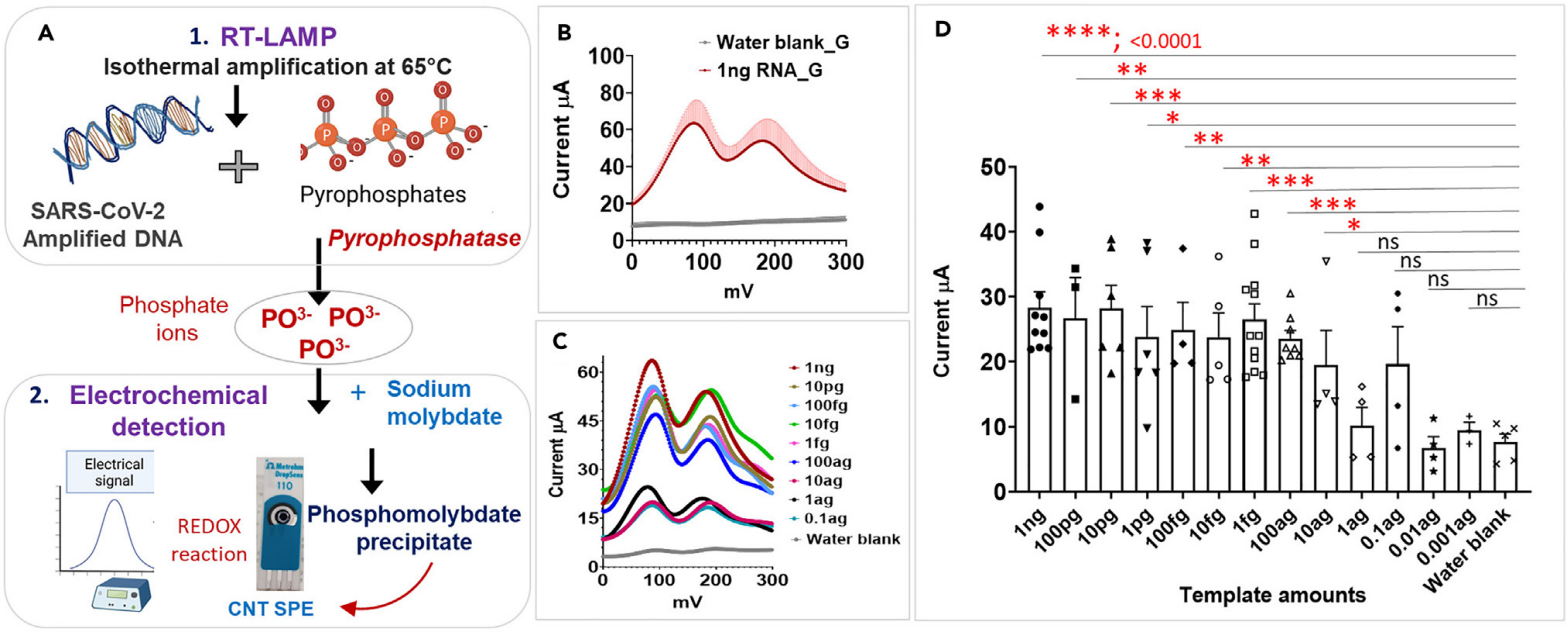
Development of SARS-CoV-2 electrochemical-RDT (A) Step-by-step procedure for electrochemical LAMP. RT-LAMP isothermal amplification is the first step conducted either in a Genie II instrument or in a heat block. Pyrophosphates generated from the LAMP reactions are cleaved to phosphate ions (PO^3−^) by pyrophosphatase used in the LAMP reaction. The second step involves mixing of LAMP reaction with sodium molybdate, incubated and dropped onto an SPE CNT electrode for CV analysis. A positive LAMP reaction will generate pyrophosphates which ultimately forms phosphomolybdate precipitate that undergoes reduction-oxidation (REDOX) reaction generating electric current (signal). (B) CV showing current profile for a positive reaction conducted with 1ng synthetic SARS-CoV2 RNA as the template and negative with water as the blank reaction (water blank_G). (C and D) Limit of detection (LOD) analysis of the electrochemical LAMP. A varying amount of RNA templates from 0 to 0.1ag were used as the template. CV analysis shows the peaks from positive reactions. Reactions with 100 and 10ag of RNA produced significantly higher currents (μA) than the negative water blank indicating the sensitivity of the electrochemical LAMP as 10ag. Values are mean ± SEM (n = 3–5). ∗ indicate statistical significance between different groups tested using unpaired t-test with Welch’s correction assuming unequal variances; p < 0.05.

A typical LAMP reaction generates pyrophosphates which is proportional to the amount of amplified nucleic acid. However, in this study we used thermostable pyrophosphatase into the LAMP reaction buffer which cleaves pyrophosphates into phosphates (Pi) during the LAMP reaction. The pyrophosphates when combined with sodium molybdate produce phosphomolybdate precipitate which undergoes redox reactions to generate an electric current under acidic conditions. The principle of an electrochemical LAMP is based on the generation of redox potential for a positive LAMP reaction.^33^ The schematic of the electrochemical LAMP workflow is shown in Figure 2A. The first step involved a LAMP reaction; isothermal incubation was performed either using a Genie II (a platform for isothermal amplification of DNA or RNA) or a simple heat block to run isothermal amplification at 65°C for 30 min. The second step involved the addition of an equal volume of sodium molybdate (prepared in deionized (DI) water) to the completed LAMP reaction followed by incubation for 10 min at room temperature. The third step involved transferring up to 10μL of LAMP + sodium molybdate mix to the carbon nanotube (CNT) screen printed electrodes (SPE) followed by drying and recording cyclic voltammetry (CV) of the sample using a potentiostat. Sulfuric acid was used as the electrolyte and the parameters for measuring CV was set as previously described.^33^ The electrochemical detection including the prior RT-LAMP step was completed within 45 min. Figure 2B shows CV graph with current (μA) plotted vs. voltage (mV) for a positive LAMP reaction demonstrating redox peaks for a positive reaction performed with SARS-CoV2 RNA (1ng) as the template. The average current calculated for a positive LAMP was significantly higher than the blank reaction (Figure 2C). The average current was ∼30 μA for a positive LAMP reaction where the isothermal amplification was conducted either in a Genie II instrument or on a heat block (Figure S2). We calculated the LOD for the electrochemical LAMP by diluting synthetic RNA from 10^−0^ to 10-^10^ (0.1attograms) in nuclease free water. The LOD of electrochemical-RDT was recorded to be 10^− to8^-fold dilution of 1ng (10attograms) which accounts for 5.29 copies of ssRNA per reaction or 5.29 × 10^3^ copies/mL (Figures 2C and 2D). Here we demonstrate that our SARS-CoV-2 electrochemical-RDT has a sensitivity of detecting as allow as 10 ag of viral DNA (5.29 copies of ssRNA per reaction) and that the sensitivity was 10-fold better than the conventional fluorescent LAMP which has a LOD of 100 ag (52.95 copies of ssRNA per reaction) (Figure 2D).

### SARS-CoV-2 detection in simulated biological samples using electrochemical-RDT

Initial experiments tested the performance of the electrochemical-RDT in synthetic samples prepared by spiking SARS-CoV-2 RNA into human serum and saliva. The results showed uniform positives in both spiked and unspiked samples indicating a false-positive rate of 100%. We hypothesized that these were caused by phosphate ions in biological fluids that interfered with electrochemical detection of phosphate ions generated in a positive, but not negative, LAMP reaction. To eliminate this problem, we introduced a sample clean up step for clinical samples using phosphate removal columns prior to the LAMP reaction; this phosphate removal step is completed within 5 min (Figures S3 and S4 and see STAR methods). We also checked background interference for samples prepared in viral transport media (VTM) and lysis buffer (see STAR methods) that were used for collection of clinical swabs from human participants (detailed discussion in the next section). We demonstrated the efficiency of commercial phosphate removal columns by using molybdenum blue spectrophotometric method for phosphate detection in samples with and without phosphate removal (Figure S4).

**Figure 3:**
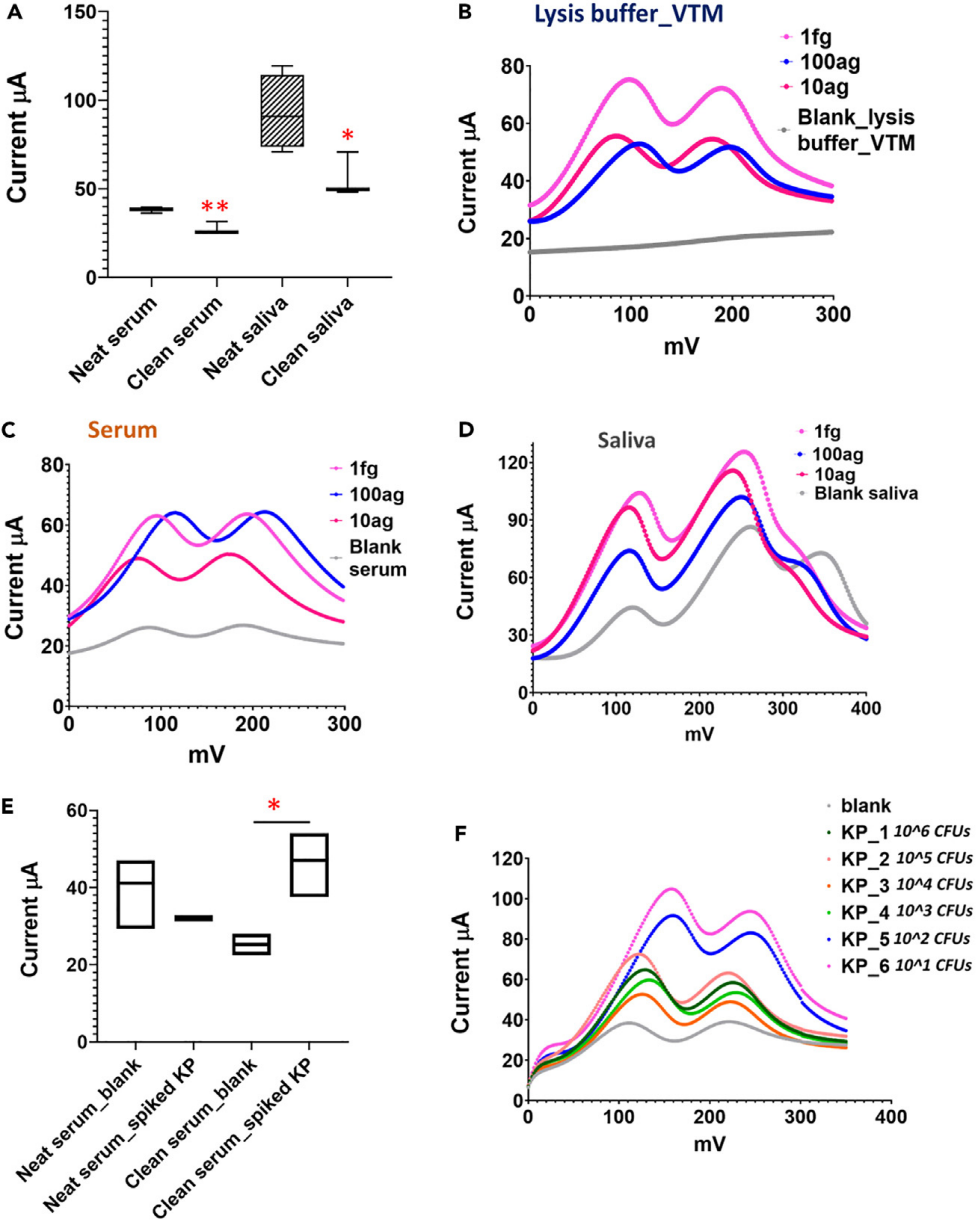
Development of electrochemical-RDT for simulated biological samples (A–D) Effect of phosphate removal on electrochemical detection of human serum and saliva samples. There was a significant reduction of background interference in neat serum and saliva samples after phosphate removal step. Values are mean ± SEM (n = 3–4). Sensitivity analysis of SARS-CoV-2 electrochemical LAMP in (B) lysis buffer VTM, (C) human serum and (D) human saliva. Neat serum and saliva were treated with phosphate removal columns to remove any background or matrix phosphates prior to LAMP and electrochemical detection. 10ag is the LOD for the electrochemical LAMP and templates 1fg, 100ag and 10ag were clearly distinguishable from blank samples. The analyses were repeated independently at least 3–4 times for the three categories of simulated samples. (E) Electrochemical detection of KP LAMP in neat and phosphate removed serum. Values are mean ± SEM (n = 3–4). (F) Sensitivity analysis of KP LAMP. Live KP with different dilutions were spiked into phosphate removed human serum. Our test was sensitive to detecting as low as 10 colony forming units. Statistical was done using one-way ANOVA and Tukey’s and unpaired t-test; ∗, p < 0.05; ∗∗, p < 0.005.

Human serum and saliva were pre-treated for phosphate removal, thus eliminating any background interference and suppression of electrochemical signal in biological samples (Figure 3A). After validation of the sample clean-up step for background phosphate removal, samples were checked for any interference that may have originated due to the components of the lysis buffer and VTM. We tested low RNA template concentrations of 1fg, 100ag and 10ag in the LAMP reactions to determine the sensitivity of positive vs. negative blank reactions. The use of lysis buffer and VTM did not affect the sensitivity of the electrochemical LAMP as shown by a clear difference in signals between the positive (lower LOD at 10ag) and negative LAMP reactions (Figure 3B). Figures 3C and 3D shows the sensitivity analysis carried out with clean serum and saliva samples that were spiked with SARS-CoV-2 RNA prepared in lysis buffer and VTM. CV analysis of these samples shows no interference with electrochemical detection and sensitivity in reactions with template as low as 10ag (Figures 3C and 3D; Figures S5A and S6A). However, the templates 1fg, 100ag, and 10ag when spiked into biological fluids serum and saliva did not demonstrate a significant concentration-dependent separation like that measured for the RNA prepared in nuclease free water (Figure 2C). We believe that this pattern could potentially be due to some minor interference from the inherent elements and ions present in the biological fluids other than phosphates which may cause the peak shifts and the non-concentration dependent pattern for the low template concentrations in serum and saliva. But these interferences are insignificant and does not affect the sensitivity of the electrochemical-RDT as there are can clear differences between the negative (blanks) and the positive samples (RNA templates in serum or saliva). In summary, we have shown that our electrochemical detection is reproducible with simulated samples prepared with serum and saliva. We checked the matrix effect of using biological fluids for fluorescent LAMP (Figures S6B and S6C). The effect of phosphate removal did not improve the sensitivity or detection efficiency of the fluorescent LAMP. This is evident from the lack of significant differences between the signal of blank reactions prepared in neat serum or phosphate-removed serum (Figure S5B). This was not the case for saliva spiked samples. The fluorescent signal detection improved after addition of phosphate removal step from saliva (Figure S6B).

Additionally, we checked the versatility of our electrochemical-RDT for the detection of *Klebsiella pneumoniae* spiked into human serum. This serum was treated for phosphate removal as discussed for SARS-CoV-2 test. We targeted *K. pneumoniae yhaI* gene as described in Poirier et al., 2022.^16^ The primer sequences for *K. pneumoniae* (KP) LAMP are included in Table S1. Live bacteria were spiked into human serum followed by cell lysis and DNA extraction (see STAR methods). Figure 3E shows the electrochemical detection of KP spiked in neat vs. phosphate removed (clean) serum. There was a significant increase in sensitivity of KP detection in clean serum. The KP electrochemical assay was sensitive to detecting as low as 10 viable colony forming units per mL (Figure 3F).

### SARS-CoV-2 detection in clinical samples using electrochemical-RDT

**Figure 4:**
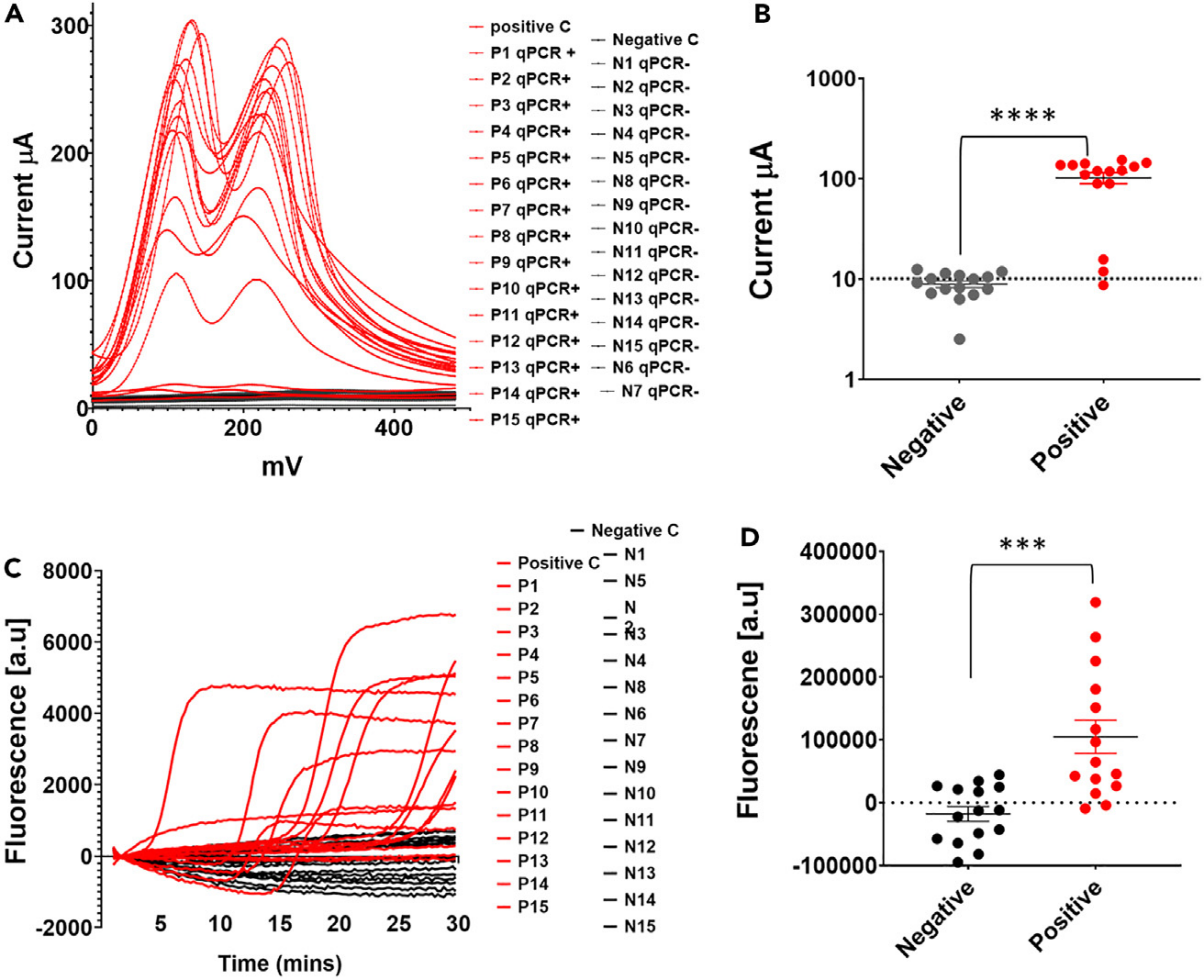
SARS-CoV-2 detection in clinical samples using electrochemical-RDT (A) Electrochemical-RDT based detection of positive (red) and negative (blank) samples. 30 clinical swabs were tested, out of which 14 were positive for SARS-CoV-2 in the electrochemical test. These samples were also positive for RT-qPCR test. (B) Average current (μA) for SARS-CoV-2 RT-qPCR identified positive and negative samples. (C) Fluorescent LAMP detection of positive (red) and negative (blank) samples. 11 out of 30 tested samples were positive for SARS-CoV-2 using fluorescent LAMP. (D) Average fluorescence for SARS-CoV-2 positive and negative samples identified by RT-qPCR. Statistical was done using unpaired t-test with Welch’s correction; ∗∗∗, p < 0.0005; ∗∗∗∗, p < 0.00005. P1-P15 are swab samples tested positive by RT-qPCR (shown in red) and N1-N15 are samples tested negative by RT-qPCR.

We next validated our electrochemical-RDT test using clinical saliva and nasal-pharyngeal swab samples. RNA was extracted from clinical samples using one-step lysis buffer extraction (see STAR methods). RT-qPCR, which is the gold standard widely used test for COVID-19 diagnostics was used as the reference test to check the electrochemical LAMP sensitivity in clinical samples. RT-qPCR was performed for 30 individual samples; 15 were identified as positives and 15 were identified as negatives. Figures 4A–4D shows the electrochemical and fluorescence detection of SARS-CoV-2 in human clinical swab samples. The Ct values of RT-qPCR tested samples and their comparison to electrochemical and fluorescence LAMP is shown in Table S2. The electrochemical-RDT detected 14 positive samples out of 30 tested (Figure 4A). The fluorescent RT-LAMP test detected 11 positives out of 30 samples tested (Figure 4B). These results demonstrate a higher sensitivity of the electrochemical assay (detected 14 out of 15 positive samples: 93.33%) for detecting SARS-CoV-2 in clinical samples than the fluorescent LAMP (detected 11 positives out of 15 positives; 73.3%).

## Discussion

Rapid and accurate diagnostics are paramount for the management and control of infectious disease epidemics and pandemics. Several molecular diagnostic platforms have been developed to tackle the recent COVID-19 pandemic.^19^^,^^34^ In this study, we have developed a new electrochemical-RDT that provides a rapid and sensitive detection of SARS-CoV-2 in spiked biological fluids (serum and saliva) and in clinical swab samples. Our method includes a sample pre-treatment step that removes background phosphates in biological samples that would otherwise interfere with electrochemical detection.

RT-LAMP is a molecular diagnostic technique and an alternative to qRT-PCR providing faster detection of transcripts of target pathogens using constant amplification and requiring fewer and less expensive resources.^18^^,^^24^ Conventional RT-LAMP detection techniques using turbidity, fluorometry and colorimetry are associated with challenges such as false negatives arising from low template material, false positives due to cross contamination or carryovers and the need for specialized equipment as those required for fluorescence detection.^15^^,^^19^ Several SARS-CoV-2 RT-LAMP end-detection techniques have been developed and optimized to improve accuracy and sensitivity and to provide platforms for POC applications. Cod-UNG-rRT-LAMP assay improved the sensitivity of the real time fluorescence, colorimetric and turbidity end-detection with sensitivity of detecting 2–10 copies/μL or 8 copies per reaction.^15^ However, this LAMP method used four different incubation temperatures which may add to the complexity as well as the affordability of a POC design. CRISPR-based techniques have been combined with RT-LAMP for SARS-CoV-2 detection such as the iSCAN (*in vitro* Specific CRISPR-based Assay for Nucleic acids detection) and DETECTR using cas12 one-step or two-step reactions.^28^^,^^35^ A two-step reaction assay requires addition of Cas proteins after the LAMP reaction which poses cross contamination risks and false positives due to the opening of LAMP reaction tubes and longer incubation times. A one-step system suffers from low sensitivity as these methods usually require a lower incubation temperature for the Cas proteins compared to the typical LAMP reaction set between 62°C and 65°C, thus requiring optimizations for application to POC testing.^28^^,^^35^^,^^36^ Another CRISPR/cas12a based surface plasmon resonance platform, MOPCS ((methodologies of photonic CRISPR sensing) has been developed for SARS-CoV-2 variant detection; this technique requires no DNA amplification for detection.^37^ Our electrochemical-RDT developed in this study is designed for detection of SARS-CoV-2 through amplification of the M gene and consequent redox signal generated from pyrophosphate-molybdate reaction in virus positive samples. Our assay has an advantage over the Cod-UNG-rRT-LAMP^11^ and CRISPR-based assays^24^^,^^30^ in avoiding multiple temperature-specific incubation steps. MOPCS, the CRISPR/cas12a based photonic detection requires multiple incubation steps to prepare chip and solutions for surface plasmon resonance measurements and the suitability of detection in crude biological fluids has not been tested, thus requiring further optimizations for POC settings.^37^ Our assay requires only the RT-LAMP step to be done at 65°C and the subsequent electrochemical reaction can be done at room temperature thus making our test suitable for POC settings. The electrochemical-RDT provided rapid and accurate detection and reduced false positive contaminations when compared to the conventional fluorescent RT-LAMP. The specificity and sensitivity were better than the conventional RT-LAMP fluorometric assay and equivalent to other techniques such as the CRISPR-based MOPCS platform (93.33% detection accuracy validated using saliva and nasal-pharyngeal swabs).

Multiple upconversion nanomaterial-based biosensors have been developed for COVID-19 such as the Förster resonance energy transfer (FRET) based gold nanorods and nanoparticles detection of SARS-CoV-2 spike (S) protein and a smart phone-controlled luminescence-based diagnostic platform targeting N gene of SARS-CoV-2.^38^^,^^39^^,^^40^ These platforms demonstrated sensitivity and specificity when tested in RNA extracted from COVID-19 positive samples but the suitability of this nanomaterial-based biosensors for crude biological samples needs to be tested. The key considerations in nanoparticle-based bioassays are the choice of best nanoparticles for detection, their synthesis and surface modifications, which may be an expensive and time-consuming process. The electrochemical-RDT developed in this study has advantages over these biosensors as the detection relies on using inexpensive and disposable carbon screen printed electrodes and the test was validated with crude saliva and serum samples.

Several researchers developed electrochemical-based diagnostics for bacterial and viral pathogens and highlighted their potential for development of efficient POCs.^33^^,^^41^^,^^42^ Reliable and efficient POC devices require simple protocol, application and low-cost implementation in various laboratory and diagnostic settings. Electrochemical-based sensors using SPE provides effective and low-cost detection of targets. SPE surfaces can be modified to improve sensitivities, such as the modification of SPE surfaces with MXene Ti3C2Tx coating matrix has been applied to detect acetaminophen and isoniazid in biological samples.^43^ Our electrochemical test uses RT-LAMP to detect SARS-CoV-2 M gene target and provides advantages over conventional PCR avoiding the need for expensive equipment, multiple processing steps, and specially trained personnel. RT-LAMP and other isothermal amplification techniques such as rolling circle amplification (RCA)-based electrochemical sensors has been developed for the detection of SARS-CoV-2; however, these assays involve multi-step amplification steps and required lengthier time of up to 2 h to complete from sample to test.^44^^,^^45^ Our electrochemical-RDT provides CV as a rapid readout for positive LAMP products within 45 min and with significantly higher sensitivity and specificity compared to the conventional fluorescent RT-LAMP. Therefore, our electrochemical-RDT has a better suitability for the development of a POC test.

Electrochemical LAMP-based assays have so far been validated only for extracted bacterial or viral nucleic acid samples and not for crude biological fluids. The electrochemical LAMP based detection of *Nosema bombycis* (a pathogen of silkworms) developed by Xie et al., 2015 was validated only for extracted DNA samples.^33^ The detection of hepatitis B virus using electrochemical LAMP was validated for viral DNA extracted from serum samples.^41^ Here we improvised our electrochemical-RDT to reliably detect SARS-CoV-2 in biological fluids such as serum and saliva which contained phosphates. The inherent phosphates in biological fluids lead to interference and matrix effects in the electrochemical assay that relied on the redox reaction between pyrophosphates (generated from positive LAMP reaction) and molybdate. To eliminate background interference, we optimised column-based phosphate removal for biological sample clean up prior to LAMP-based electrochemical detection.

We tested the sensitivity and specificity of our electrochemical-RDT using viral RNA samples prepared in nuclease free water, RNA spiked into human serum and saliva and clinical saliva and nasal-pharyngeal swab samples. The LOD of our electrochemical assay was 5.29 copies/μL, which is similar to or lower than several SARS-CoV-2 diagnostic assays (such as the RT-PCR CovidNudge, the colorimetric and fluorescent LAMP, LAMP coupled to the nanopore sequencing assay LamPORE).^17^^,^^18^^,^^27^^,^^29^^,^^46^ RT-LAMP assays have been developed for detecting SARS-CoV-2 variants and for performing multiplex assays.^31^^,^^47^^,^^48^ As electrochemical-RDT uses LAMP, this test could easily be extended for the detection of multiple targets and variants/lineages with improved accuracy and sensitivity compared to the conventional fluorescent RT-LAMP.^49^

The electrochemical-RDT was 100% specific to SARS-CoV-2 when cross reactivity was checked against a panel of 21 bacterial and viral respiratory pathogens that included adenovirus type 6, *Bordetella pertussis* and *Bordetella parapertussis*, *Chlamydia pneumoniae*, coronaviruses 229E HKU1, NL63, OC43 surrogates, human metapneumovirus surrogate (hMPV), human rhinovirus, influenza A and B, *Mycoplasma pneumoniae*, parainfluenza viruses, respiratory syncytial virus and avian coronavirus infectious bronchitis virus (IBV Beau-R). When applied to detecting SARS-CoV-2 in clinical swab samples, the electrochemical-RDT demonstrated 100% specificity and 93.33% sensitivity in samples that were previously tested as positive or negative using RT-qPCR. The electrochemical-RDT demonstrated higher sensitivity (∼1.2-fold) than fluorescent RT-LAMP when tested with the same clinical samples. We extended our test to *K. pneumoniae* detection spiked into human serum that was pre-treated for phosphate removal. The test detected as low as 10 colony forming units per mL, demonstrating the applicability of our electrochemical-RDT for other microbes.

In conclusion, we have developed a rapid, sensitive, specific, and inexpensive RT-LAMP based electrochemical-RDT targeting the M gene of SARS-CoV-2. The test CV as a readout for detection of SARS-CoV-2 rapidly (within 45 min) and reliably in simulated biological and clinical samples. The test is highly sensitive compared to conventional RT-LAMP in detecting SARS-CoV-2 RNA as low as 5.29 copies/μL and demonstrated 100% specificity and 93.33% sensitivity in extracted clinical swab samples. Our assay was optimized for biological fluids such as serum and saliva by including a phosphate removal step to reduce background interference from phosphates in human fluids. We extended our assay to detecting *K. pneumoniae* in human serum, demonstrating the suitability of the platform for both bacterial and viral diagnostics. The conversion of the LAMP signal to a simple electric current provides the opportunity of further miniaturizing of the test onto a lab-on-a-chip platform that could be operated by a smartphone and could be easily integrated with healthcare provisions.

### Limitations of the study

The electrochemical-RDT is currently limited to detecting one gene target and further work is needed to expand detection for multiple targets and variants/lineages of SARS-CoV-2. The test was validated only for SARS-CoV-2 and *K. pneumoniae* so, there is further scope to extend the test for detection of other microbial species.

## STAR★Methods

### Key resources table

**Table:** 

REAGENT or RESOURCE	SOURCE	IDENTIFIER
**Chemicals, peptides, and recombinant proteins**		

Isothermal amplification buffer	New England Biolabs	B0537S
6mM MgSO4	New England Biolabs	B1003
1.4mM dioxyribonucleotides dNTPs	New England Biolabs	N0447S
320U/mL Bst 2.0 DNA polymerase	New England Biolabs	M0537S
7500U/mL warmStart® RTx reverse transcriptase (NEB),	New England Biolabs	M0380S
2000U/mL thermostable inorganic pyrophosphatase	New England Biolabs	M0296S
SYBR green nucleic acid stain	Fisher scientific UK	10710004
nuclease free water	New England Biolabs	B1500S
sodium molybdate	Merck	243655
Screen-printed carbon nanotube (CNT) electrodes (110-D)	Metrohm Dropsens	DRP-110CNTU50
micro phosphate removal columns	ProFoldin	MPR020
Igepal	Merck	I8896
NaCl,	Merck	S9888
Tris-HCl (pH 7.4),	Merck	T2194
Guanidine-HCl	Merck	PHG0006
RNeasy kit	QIAgen	74004
Real-Time RT-PCR Diagnostic Panel containing the 2019-nCoV_N1, 2019-nCoV_N2 and Human RNase P combined primers and probes mix	(Centers for Disease Control and Prevention, Division of Viral, Atlanta USA),	N/A
Sulfuric acid	Merck	258105
L-Ascorbic acid	Merck	A4403
Ammonium molybdate tetrahydrate	Merck	A7302
Potassium antimony tartrate trihydrate	Merck	383376
Potassium phosphate monobasic (0.1M)	Merck	P0662
Respiratory pathogens control panel DNA	Microbiologics Minnesota, USA	N/A

**Experimental models: Organisms/strains**		

*Klebsiella Pneumoniae* KPC producing strain NCTC13809	Center for Disease Control, Atlanta, GA, USA	SAP 3874

**Oligonucleotides**		

5′-TAATACGACTCACTATAGTAATCAGACAAGG AACTGATTA-3′ 5′-CGAAGGTGTGACTTCCATG-3′	This work; Merck	Primers for SARS-CoV-2 membrane (M) protein synthetic RNA synthesis
F3: ATGTATTGATCGCCACCG B3: GAGCCAGCGAAATAATTGC FIP: TCAACAGCAGCCAGAGCGCTCAATACGG CATCCTCAG BIP: CGGCGGATGCATGATATCGGAAACCACG GAATGATAACCC LF: GGCAAAGGGAATGACAACAAA LB: CCGGGATCTGGTTTGTGT	This work; Merck	LAMP primers for amplification of M gene
F3: ATGTATTGATCGCCACCG B3: GAGCCAGCGAAATAATTGC FIP: TCAACAGCAGCCAGAGCGCTCAATACGGCAT CCTCAG BIP: CGGCGGATGCATGATATCGGAAACCACGGAA TGATAACCC LF: GGCAAAGGGAATGACAACAAA LB: CCGGGATCTGGTTTGTGT	Merck	LAMP primer sets designed for this study, targeting the *K. pneumoniae* Inner membrane protein (*yhaI*)

**Recombinant DNA**		

A synthetic plasmid encoding the SARS-CoV-2 M gene (Wuhan-Hu-1 isolate, GenBank accession number NC_045512.2)	Integrated DNA Technologies	N/A
Membrane protein (M) gene synthetic RNA	This work	N/A

**Software and algorithms**		

GISAID (27-03-2020)	Database	https://gisaid.org
BLAST v2.12	National center for biotechnology information	
MUSCLE v3.8	European Bioinformatics Institute	
LAMP Designer tool	Optigene	
GAMRY EChem analyst software	Gamry instruments	
Prism 8.0	Graphpad	

**Other**		

Genie® II instrument (suitable for real-time fluorescence measurement)	Optigene	
GAMRY potentiostat Interface BETA-1000E	Gamry instruments	

### Resource availability

#### Lead contact

Further information and requests for resources and reagents should be directed to and will be fulfilled by the lead contact, Johnjoe McFadden (j.mcfadden@surrey.ac.uk).

#### Materials availability

All unique/stable reagents generated in this study are available from the lead contact with completed materials transfer agreement.

### Experimental model and subject details

SARS-CoV-2 synthetic RNA generated using a plasmid encoding the SARS-CoV-2 M gene (Wuhan-Hu-1 isolate, GenBank accession number NC_045512.2) was used as the template for the LAMP assays. M gene transcript RNAs were transcribed *in vitro* using the T7 RNA polymerase run-off reactions from PCR products containing the T7 polymerase promoter sequence and purified. *Klebsiella Pneumoniae* KPC producing strain NCTC13809 was used for *yhaI* LAMP assays. *K. pneumoniae* strains were cultured aerobically at 37°C for 16 h on Klebsiella selective agar and Luria–Bertani agar.^16^

#### Ethics statement

The ethical approvals for collection and use of clinical swabs for electrochemical and fluorescent LAMP tests were obtained from the UKECA-recognised Research Ethics (IRAS project ID 283201) and NHS Research and Ethics Committee (REC) (20/EE/0125).

### Method details

#### Identification of suitable gene targets for detection of SARS-CoV-2 by LAMP

We targeted membrane (M) glycoprotein which is an abundant structural protein in Coronaviruses and plays an important role in RNA packaging.^2^^,^^4^ 1654 SARS-CoV-2 genomes were downloaded from GISAID (27-03-2020). These genomes represented all sequenced SARS-CoV-2 genomes at the time of access. Nucleotide variation of each ORF was determined using BLAST v2.12 relative to the Wuhan-Hu-1 reference sequence (NC045512). Alignment of representative ORFs exhibiting sequence variation was performed using MUSCLE v3.8 and showed that the N-gene, ORF7a, and M-gene encoding structural proteins had a high degree of nucleotide conservation. LAMP primers to specifically target conserved regions of each of these genes were designed using LAMP Designer (Optigene, UK). Comparison of the N-gene, ORF7a, and M-gene to the corresponding structural genes in representative SARS, MERS, and bat SARS-like coronavirus genomes to evaluate potential cross-reactivity was performed using MUSCLE v3.8.

#### SARS-CoV-2 membrane (M) protein synthetic RNA synthesis

A synthetic plasmid encoding the SARS-CoV-2 M gene (Wuhan-Hu-1 isolate, GenBank accession number NC_045512.2) was purchased from Integrated DNA Technologies. The M gene was first PCR amplified to introduce a T7 promoter in the 5′UTR using the following primers 5′-TAATACGACTCACTATAGTAATCAGACAAGGAACTGATTA-3′ and 5′-CGAAGGTGTGACTTCCATG-3’. M gene transcript RNAs were then directly transcribed *in vitro* using the T7 RNA polymerase run-off reactions from PCR products containing the T7 polymerase promoter sequence and purified on a 7 m urea and 10–20% polyacrylamide gel, eluted by diffusion, ethanol precipitated, and quantified using a spectrophotometer.^50^

#### Reverse transcriptase (RT)-loop-mediated isothermal amplification (LAMP)

LAMP reaction buffer was designed to achieve maximum amplification along with minimum interference in electrochemical detection. The buffer composition for a 25μL reaction includes 1X isothermal amplification (buffer containing 20mM Tris-HCl, 10mM ammonium sulfate (NH_4_)_2_SO_4_, 50mM potassium chloride KCl, 2mM magnesium sulfate MgSO_4_, 0.1% Tween 20, (pH 8.8 @ 25°C)) (NEB), 6mM MgSO_4_ (NEB), 1.4mM dioxyribonucleotides dNTPs (NEB), 1.6μM FIP + BIP primers (Merck), 0.2μM F3/B3 primers (Merck), 0.4μM loop F/B primers (Merck), 320U/mL Bst 2.0 DNA polymerase (NEB), 7500U/mL warmStart RTx reverse transcriptase (NEB), 2000U/mL thermostable inorganic pyrophosphatase (NEB). SYBR green nucleic acid stain was added at 0.1X final concentration for fluorometric detection and finally, nuclease free water to make up the total volume of the LAMP reaction to 25μL reaction. 5μL of template RNA (in a range of concentrations from 1 ng/μL to 0.1 ag/μL) or nuclease free water as blank were added to the reaction mix. The reaction was spun briefly to avoid any air bubbles. The reactions were incubated at 65°C for 30 min using a Genie II instrument (suitable for real-time fluorescence measurement) or a heat block for isothermal amplification. For determining the RNA copy number/μL or copy(s)/mL in the limit of detection assays, RNA concentration was first measured using spectrophotometry and the copy number of standard RNA was calculated using the standard formula: template X g/μL RNA/[transcript length in nucleotides x 340]) x 6.022 x 1023 = Y RNA/μL.

**Table:** 

Components	Added to 25 μL reaction
10X isothermal amplification buffer	1X
MgSO4	5 mM
DMSO	2.50%
dNTPs	2.5 mM
SYBR green stain	0.1X
Bst polymerase	1U
Phosphatase	1U
transcriptase	7.5U
Nuclease free water	Required to make upto 25 μL

#### Cross specificity test for SARS-CoV-2 RT-LAMP

The SARS-CoV-2 LAMP primers set was validated for its analytical specificity by testing cross-reactivity against other respiratory pathogens: Adenovirus Type 6, *Bordetella pertussis* and *parapertussis, Chlamydia pneumoniae*, Coronaviruses 229E HKU1, NL63, OC43 surrogates, Human Metapneumovirus surrogate, Human Rhinovirus, Influenza A (subtypes H1, H1-2009 and H3) and Influenza B, *Mycoplasma pneumoniae*, Parainfluenza Viruses (1, 2, 3, and recombinant 4a) and Respiratory Syncytial Virus (using the Respiratory (21 Targets) Control Panel, Microbiologics Minnesota, USA) and Avian Coronavirus Infectious Bronchitis Virus (IBV Beau-R). Table S3 includes the respiratory pathogens tested. IBV Beau-R RNA from was extracted from virus stocks using RNeasy columns (QIAgen), following the manufacturer’s instructions, and including on-column DNAse treatment using an RNase-free DNase set (QIAgen).^51^

#### Electrochemical detection

Phosphomolybdate detection for electrochemical detection was done using a single step reaction. Briefly, 25μL LAMP products were mixed with 25μL of 20mM sodium molybdate and mixed by pipetting and were incubated for 10 min. Screen-printed carbon nanotube (CNT) electrodes (110-D) were purchased from Metrohm Dropsens. These electrodes have screen printed carbon working and counter electrodes with Ag/AgCl reference. CNT electrodes were rinsed with ultra-pure DI water and primed with 0.1M sodium nitrate for 3–10 cycles. 5-10μL of LAMP + sodium molybdate mix was placed on the electrode and dried in an oven at 50°C for 3–5 min. Dried electrodes were rinsed with ultra-pure DI water and connected to the GAMRY potentiostat electrode holder. Around 200μL of 0.5M sulfuric acid was dropped onto electrode to cover the reference, counter and working electrodes. CV was measured using GAMRY potentiostat Interface BETA-1000E instrument for oxidation and reduction peaks with a range of potential (V) from −500mV to 500mV. The measurement parameters as described in Table S4. Data analysis and visualisation was done using GAMRY EChem analyst software and Graphpad prism 8.0. A full CV spectrum for positive and negative electrochemical LAMP reaction is shown in Figure S7. Raw CV graphs in Figures 2, 3, and 4 were processed by selecting a particular region of the oxidation peaks from 0 to 400mV potential to compare the signals between various samples. Average current was calculated across the selected region of the oxidation peaks.

#### Electrochemical detection of synthetic or simulated clinical samples

Biological fluids such as serum and saliva will inherently have phosphate ions which will interfere with the electrochemical detection. To avoid phosphate interference from serum and saliva, we performed a sample clean up step of human serum and saliva (procured from healthy donors) with micro phosphate removal columns purchased from ProFoldin. The phosphate removal step is completed within 5 min. Briefly, the pre-packed columns were spun at 13000 rpm for 1 min to set down the resin. The bottom tips and caps of the columns were removed, the columns were placed in 1.5mL eppendorfs and spun for 1 min. 200μL of sample was added to one column, spun at 1000rpm for 1 min and then at 13000 rpm for 1 min. The elute was collected in a separate Eppendorf and spun at 13000 rpm for 1 min to remove any insoluble material. Using phosphate removal step, we were able to show clear reduction in the background noise of the blank samples and improved amplification of targets spiked into the biological fluids. SARS-CoV-2 RNA was added to clean serum and saliva or live *Klebsiella Pneumoniae* were added to clean serum. Lysis buffer was added to the spiked fluids for DNA/RNA extraction which is discussed in the next section. The colony forming units per mL of *K. pleumoniae* electrochemical-RDT was calculated by diluting original culture and plating 100 μL of each dilution on Luria–Bertani agar plates in triplicates to calculate the bacterial count.^16^

#### RNA extraction using lysis buffer from clinical swabs and spiked biological fluids and qRT-PCR analysis

1X lysis buffer was buffer was prepared by mixing 0.3% Igepal, 90mM NaCl, 6mM Tris-HCl (pH 7.4), 240mM of Guanidine-HCl in molecular grade water (DEPC-treated water). All materials were purchased from Merck. The buffer was filter sterilized with a 0.22μm syringe filter and stored at −20°C until use. For RNA extraction, lysis buffer 2X was mixed with clinical saliva and nasal-pharyngeal swab samples (or the swab was immersed directly in lysis Buffer 1X) or RNA spiked serum and saliva in virus transport media (VTM) or bacterial spiked serum at a ratio of 1:1. The mixture was incubated for a minimum of 5 min at room temperature. Extracted RNA was directly used for fluorescent or electrochemical LAMP. For RT-qPCR, RNA extraction was performed on 100μL of VTM using the commercial QIAgen RNeasy kit (Qiagen, Valencia, CA, USA), according to manufacturer instructions. We used the CDC 2019-Novel Coronavirus (2019-nCoV) Real-Time RT-PCR Diagnostic Panel (Centers for Disease Control and Prevention, Division of Viral, Atlanta USA), containing the 2019-nCoV_N1, 2019-nCoV_N2 and Human RNase P combined primers and probes mix, as per the Instructions for Use (fda.gov). The 25-μL RT-qPCR reactions consisted of 12.5 μL 2X Reaction Mix, 0.2 μM of each primer, and 0.1 μM probe, 0.5 μL of SuperScript III RT/Platinum Taq Mix, and 2μL of RNA (extracted from clinical samples or synthetic). The amplification process was performed in the CFX96 Touch Real-Time PCR Detection System (BioRad Laboratories, Watford, UK), according to the cycling protocol. The amount of viral RNA in each sample was estimated by comparing the cycle threshold values (Ct) to the standard curve made by serial 10-fold serial dilutions of synthetic RNA.

#### Molybdenum blue colorimetric assay for phosphate quantitation

The molybdenum blue reaction was used for the determination of orthophosphate in LAMP reactions and neat serum and saliva samples treated with and without phosphate clean up step. The principle of this reaction involves the formation of a polyoxometallate species, a heteropoly acid, from orthophosphate and molybdate under acidic conditions, which when reduced forms an intensely colored phosphomolybdenum blue species.^52^ Working solutions of sulfuric acid (11N/5.4M), ascorbic acid (6%w/v), ammonium molybdate tetrahydrate (1.26%w/v)-potassium antimony tartrate trihydrate (0.21%w/v) and potassium phosphate monobasic (0.1M) were prepared in distilled water from concentrated stock solutions. Potassium dihydrogen phosphate (KH_2_PO_4_) standards were prepared in a range of dilutions in distilled water. Samples were diluted to 5mL with distilled water. 0.1mL of working solution of sulfuric acid, 0.4mL ammonium molybdate-potassium tartrate and 0.2mL ascorbic acid solution working solution were added to the samples. The reaction was incubated at room temperature for 5 min. 1mL of the reaction was transferred to a plastic cuvette and absorbance was measured at 650nm using a spectrophotometer.

### Quantification and statistical analysis

All experiments were performed in replicates and data are presented as mean ± standard error of the mean. Cyclic voltammograms generated using GAMRY BETA-1000E potentiostat was processed using GAMRY Echem Analyst software version 7.07 purchased from GAMRY instruments. Fluorescent LAMP data was processed using Genie Explorer software purchased from Optigene. Statistical analysis including one-way Analysis of Variance (ANOVA) and unpaired t-test with Welch’s correction were done using GraphPad prism 8.2.1 version. Data visualisation was done using Microsoft excel, GraphPad prism and GAMRY Echem Analyst software.

## Data Availability

• All data reported in this paper will be shared by the lead contact upon request. • Any additional information required to reanalyze the data reported in this paper is available from the lead contact upon request. • This paper does not report original code.
