# Supplementary material for: Development of a loop-mediated isothermal amplification (LAMP)-based electrochemical test for rapid detection of SARS-CoV-2
Source: iScience. 2023 Aug 9;26(9):107570. doi: 10.1016/j.isci.2023.107570 (PMC10470312; doi:10.1016/j.isci.2023.107570)
Supplement: Document S1. Figures S1–S7, Tables S1–S4, and Data S1 [file mmc1.pdf]

iScience, Volume *26*

Supplemental information

Development of a loop-mediated isothermal amplification (LAMP)-based electrochemical test for rapid detection of SARS-CoV-2

Khushboo Borah Slater, Muhammad Ahmad, Aurore Poirier, Ash Stott, Bianca Sica Siedler, Matthew Brownsword, Jai Mehat, Joanna Urbaniec, Nicolas Locker, Yunlong Zhao, Roberto La Ragione, S. Ravi P. Silva, and Johnjoe McFadden

Figure S1. Fluorescent LAMP assay set up, Related to Figure 1.

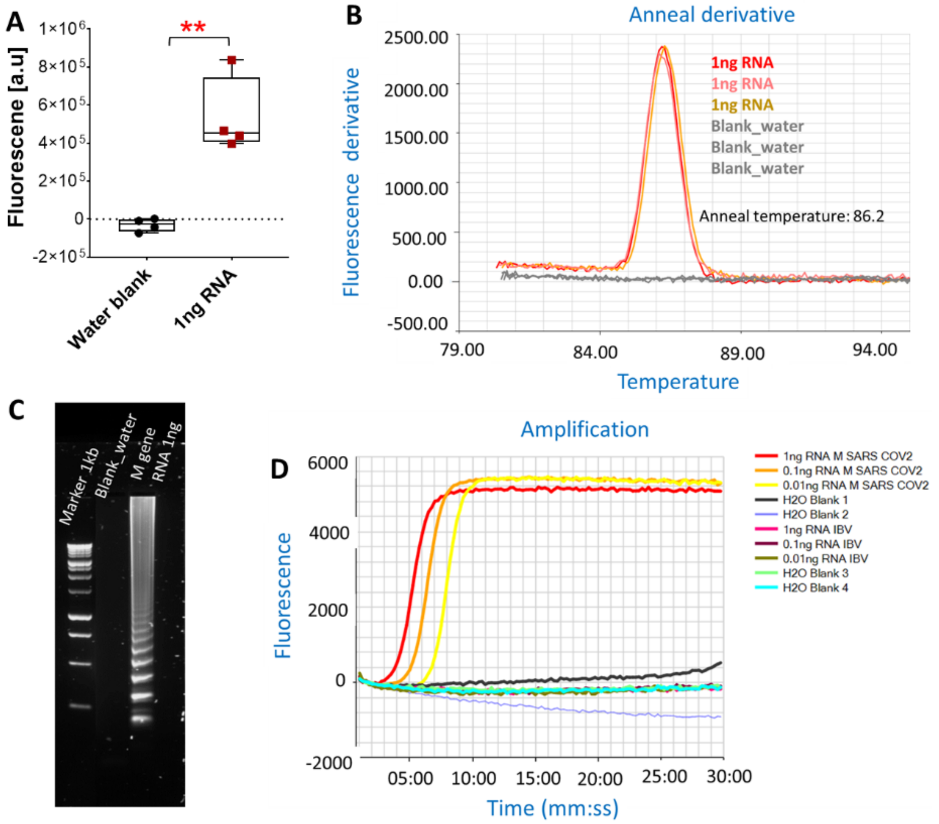

Figure S1. **Fluorescent LAMP assay set up**. A) M gene fluorescent LAMP using water blank and 1ng RNA as template. B) Anneal derivative of M gene LAMP at 86ºC. C) 1% agarose gel showing positive (1ng RNA) and negative (blank_water) LAMP reactions. D) Cross specificity test of M gene fluorescent LAMP using avian IBV coronavirus. Values are mean ± SEM (n=3). * indicate statistical significance determined using unpaired t-test with Welch's correction; p <0.05.

Figure S2. Current (μA) for positive and negative electrochemical-RDT, Related to Figure 2.

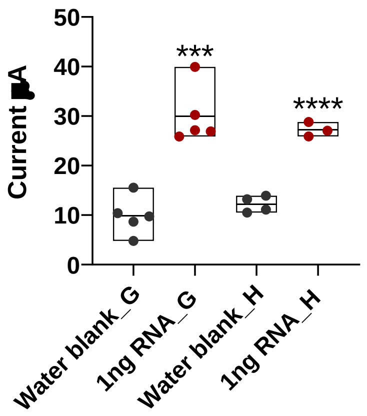

Figure S2. **Current (μA) for positive and negative electrochemical-RDT**. Values are shown for three to five independent LAMP reactions and are mean ± SEM. * indicates statistically significant differences between negative and positive LAMP reactions where the isothermal amplification was done using genie instrument (water blank_G and 1ng RNA_G) or a heat block (water blank_H and 1ng RNA_H). * indicate statistical significance determined using unpaired t-test with Welch's correction; p <0.05.

Figure S3. Electrochemical-RDT for detection of *K. Pneumoniae*, Related to Figure 3.

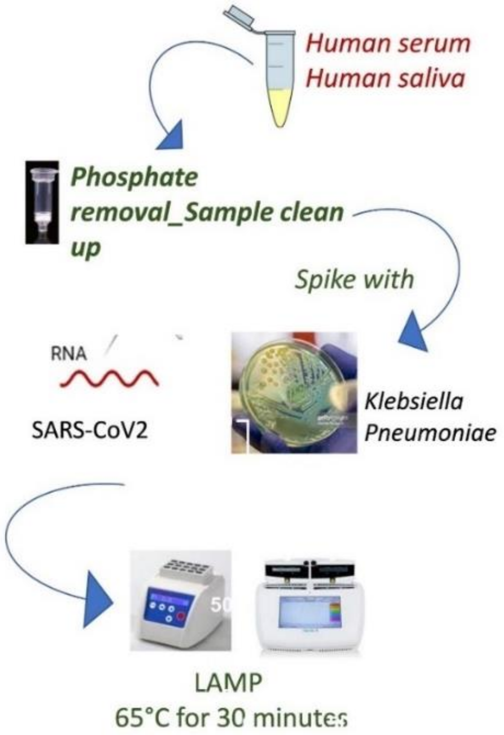

Figure S3. **Electrochemical-RDT for detection of *K. Pneumoniae***. Outline of steps in electrochemical-RDT of SARS-CoV-2 and KP in simulated biological samples prepared with human serum and saliva.

Figure S4. Molybdenum blue phosphate assay, Related to Figure 3.

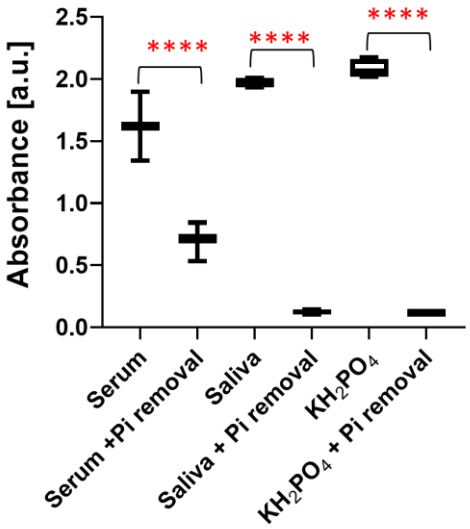

Figure S4. **Molybdenum blue phosphate assay**. Molybdenum blue phosphate detection in human serum, human saliva, and standard potassium dihydrogen phosphate KH_2PO_4 solution. * Indicate statistical significance determined using unpaired t-test with Welch's correction; $p < 0.05$. ****, $p < 0.00005$.

Figure S5. Electrochemical-RDT for detecting SARS-CoV-2 spiked in serum, Related to Figure 3.

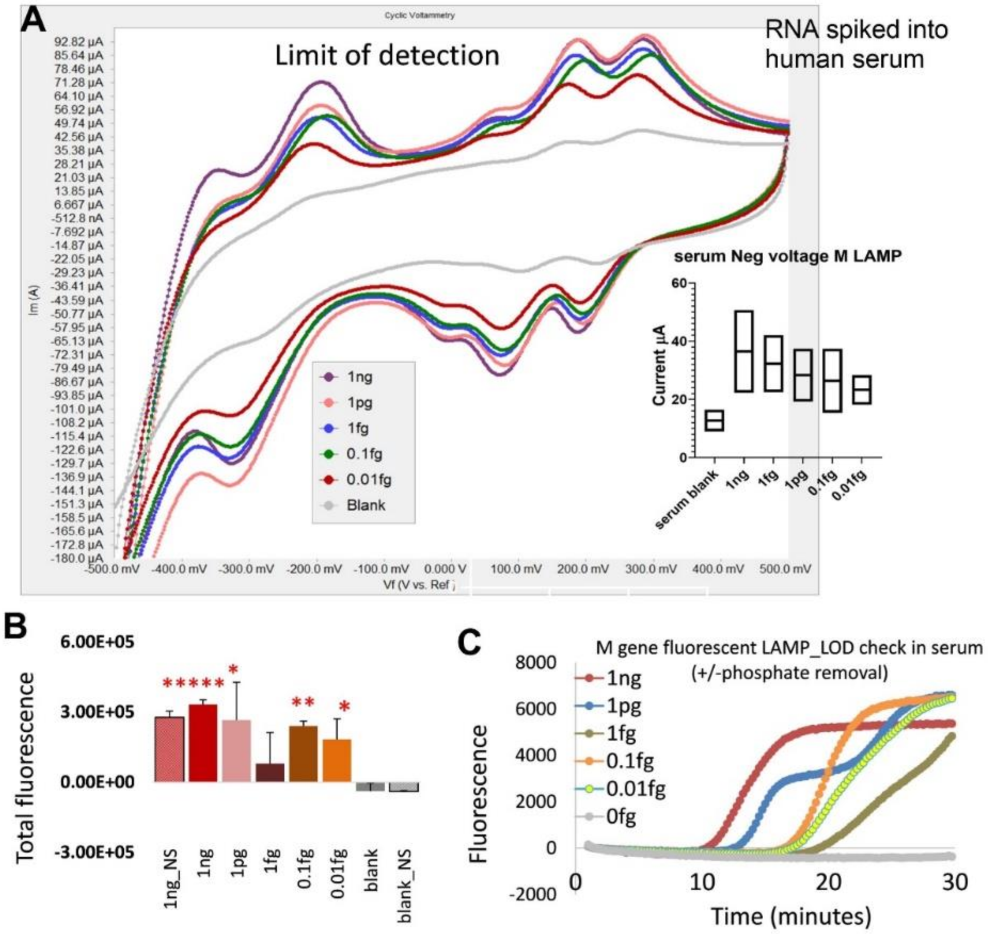

Figure S5. **Electrochemical-RDT for detecting SARS-CoV-2 spiked in serum**. A) Sensitivity analysis of electrochemical-RDT for detecting SARS-CoV-2 in human serum spiked with RNA. Cyclic voltammograms showing current peaks generated at various positive and negative potentials for the positive samples and negative serum blank sample. The positive reactions show a distinct profile from the negative and higher currents (also see inset in A). Profiles are shown for one of the three individual analyses. B) Fluorescent SARS-CoV-2 LAMP showing the effect of phosphate removal during sample preparation. 1ng_NS is 1ng RNA spiked into neat serum and shows a reduced signal as compared to the 1ng which is RNA spiked into serum after phosphate removal step. There were no statistically significant differences between the neat serum (NS) and phosphate-removed serum blanks. C) Limit of detection analysis in reactions prepared with varying amounts of RNA as the template spiked into phosphate-removed serum. Values are mean ± SEM (n=3). * Indicate statistical significance determined using unpaired t-test with Welch’s correction; $p < 0.05$.

Figure S6. Electrochemical-RDT for detecting SARS-CoV-2 spiked in saliva, Related to Figure 3.

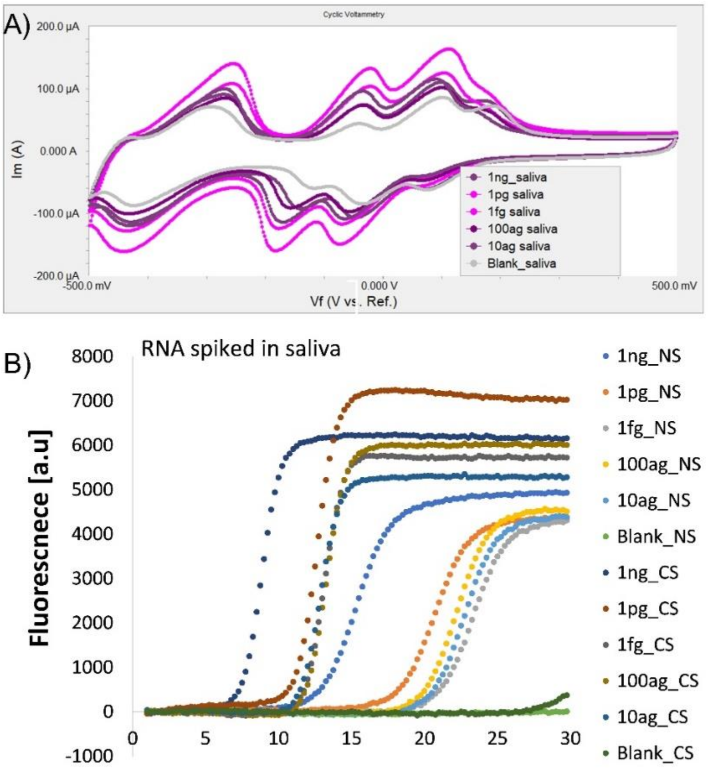

Figure S6. **Electrochemical-RDT for detecting SARS-CoV-2 spiked in saliva**. A) Sensitivity analysis of electrochemical-RDT for detecting SARS-CoV-2 in human saliva spiked with RNA. Cyclic voltammograms showing current peaks generated at various positive and negative potentials for the positive samples and negative serum blank sample. The positive reactions show a distinct profile from the negative and higher currents. B) Fluorescent SARS-CoV-2 LAMP showing the effect of phosphate removal during sample preparation. NS is neat saliva and CS stands for saliva treated with phosphate removal step. Limit of detection analysis in reactions prepared with varying amounts of RNA as the template spiked into phosphate-removed saliva and neat saliva. The phosphate removal from saliva improves the fluorescent detection. Electrochemical and fluorescent profiles are shown for one of the three individual electrochemical analyses.

Figure S7. Cyclic voltammogram of positive and negative electrochemical-RDT, Related to Star Methods.

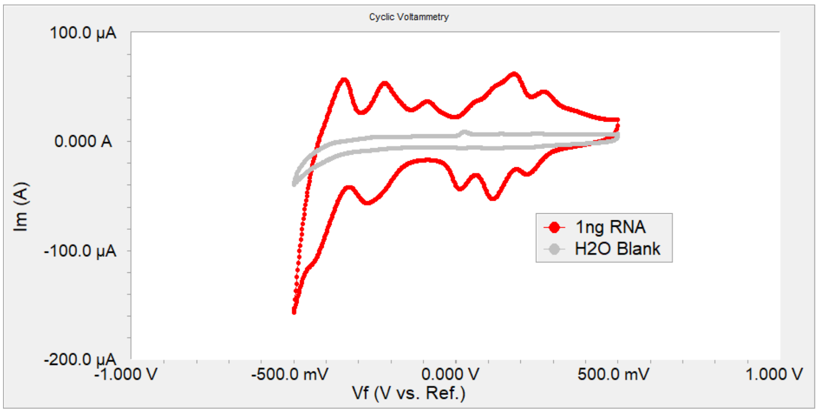

Figure S7. **Cyclic voltammogram of positive and negative electrochemical-RDT**. Cyclic voltammogram spectra of positive (+ 1ng RNA as the template) and negative (no template, + H_2O as the blank) measured using GAMRY interface BETA-1000E potentiostat.

Table S1: **Primers for M gene of SARS-CoV-2**, Related to Figure 1.

Name	Primer
F3	ATGTATTGATCGCCACCG
B3	GAGCCAGCGAAATAATTGC
FIP	TCAACAGCAGCCAGAGCGCTCAATACGGCATCCTCAG
BIP	CGGCGGATGCATGATATCGGAAACCACGGAATGATAACCC
LF	GGCAAAGGGAATGACAACAAA
LB	CCGGGATCTGGTTTGTGT
Product	Sequence Length:249

Table S2. **SARS-CoV-2 detection in clinical swabs using RT-qPCR, electrochemical and fluorescent LAMP**, Related to Figure 4.

Sample	RT-qPCR Ct values	RT-qPCR Ct values	RT-qPCR Ct values	RT-qPCR test	Electrochemical LAMP	Fluorescent LAMP
	N1	N2	RNaseP			
S1	n.d.	n.d.	32.66	Negative	Negative	Negative
S2	n.d.	n.d.	30.38	Negative	Negative	Negative
S3	n.d.	n.d.	27.78	Negative	Negative	Negative
S4	n.d.	n.d.	32.19	Negative	Negative	Negative
S5	n.d.	n.d.	29.14	Negative	Negative	Negative
S6	n.d.	n.d.	32.12	Negative	Negative	Negative
S7	n.d.	n.d.	31.63	Negative	Negative	Negative
S8	n.d.	n.d.	28.76	Negative	Negative	Negative
S9	n.d.	n.d.	32.66	Negative	Negative	Negative
S10	n.d.	n.d.	30.45	Negative	Negative	Negative
S11	n.d.	n.d.	34.18	Negative	Negative	Negative
S12	n.d.	n.d.	29.35	Negative	Negative	Negative
S13	25.19192	25.54134	28.52227	Positive	Positive	Positive
S14	32.31	30.9209	28.94717	Positive	Positive	Positive
S15	37.21	36.74755	29.57912	Positive	Positive	Negative
S16	27.11	25.9	28.54	Positive	Positive	Positive
S17	34.35	33.08	27.38	Positive	Positive	Negative
S18	28.51656	31.58848	30.01275	Positive	Positive	Negative
S19	30.05011	30.11525	25.3321	Positive	Positive	Positive
S20	24.41116	25.84249	29.05945	Positive	Positive	Positive
S21	23.10206	29.09658	24.57329	Positive	Positive	Positive
S22	n.d.	n.d.	30.81689	Negative	Negative	Negative
S23	22.00351	27.73081	23.5405	Positive	Positive	Positive
S24	20.31274	23.53867	30.55743	Positive	Negative	Positive
S25	36.96	38.45699	37.04	Positive	Positive	Negative

S26	31.1	33.36	33.98062	Positive	Positive	Positive
S27	33.3	31.81	28.19	Positive	Positive	Positive
S28	n.d.	n.d.	30.38075	Negative	Negative	Negative
S29	26.50757	32.32739	25.46112	Positive	Positive	Positive
S30	n.d.	n.d.	29.2012	Negative	Negative	Negative

For RT-qPCR RNAse P is a human gene used as an internal control to ensure that the RNA extraction worked well. N1 and N2 are two regions of the SARS-CoV-2 Nucleocapsid gene. The negative samples had only internal control RNAseP values, and the positive samples had N1 and N2 in addition to the RNAaseP. Electrochemical and fluorescent LAMP targeting M gene detected positive and negative samples which are shown alongside the RT-qPCR tests.

Table S3: **Respiratory pathogen panel used for testing primer specificity**, Related to Figure 1.

1. Adenovirus Type 6
2. Bordetella parapertussis
3. Bordetella pertussis
4. Chlamydophila pneumoniae
5. Coronavirus 229E
6. Coronavirus HKU1 surrogate
7. Coronavirus NL63 surrogate
8. Coronavirus OC43 surrogate Strain 1 and Coronavirus OC43 surrogate Strain 2
9. Human Metapneumovirus surrogate
10. Human Rhinovirus
11. Influenza A
12. Influenza A subtype H1
13. Influenza A subtype H1-2009
14. Influenza A subtype H3
15. Influenza B
16. Mycoplasma pneumoniae
17. Parainfluenza Virus 1
18. Parainfluenza Virus 2
19. Parainfluenza Virus 3
20. Parainfluenza Virus 4a surrogate
21. Respiratory Syncytial Virus

Table S4. **Measurement parameters for cyclic voltammetry**, Related to Star methods.

**Parameters**	**Values**
Initial E(v)	0 vs. Eref
Scan limit 1(V)	0.5 vs. Eref
scan limit 2(V)	-0.5 vs. Eref
Final E(v)	0.2
scan rate (mv/s)	100
Step size	2mV
Sample area (cm^2)	1
Equilibration time (seconds)	5
I/E range mode	Fixed I/E range
Maximum current (mA)	0.5

Dataset 1. M membrane glycoprotein [Severe acute respiratory syndrome coronavirus 2] sequence, Related to Figure 1 and Table 1.

https://www.ncbi.nlm.nih.gov/gene/43740571

ATGGCAGATTCCAACGGTACTATTACCGTTGAAGAGCTTAAAAAGCTCCTTGAACAATGGAACCTAGTAA
TAGGTTTCCTATTCCTTACATGGATTTGTCTTCTACAATTTGCCTATGCCAACAGGAATAGGTTTTTGTA
TATAATTAAGTTAATTTTCCTCTGGCTGTTATGGCCAGTAACTTTAGCTTGTTTTGTGCTTGCTGCTGTT
TACAGAATAAATTGGATCACCGGTGGAATTGCTATCGCAATGGCTTGTCTTGTAGGCTTGATGTGGCTCA
GCTACTTCATTGCTTCTTTCAGACTGTTTGCGCGTACGCGTTCCATGTGGTCATTCAATCCAGAAACTAA
CATTCTTCTCAACGTGCCACTCCATGGCACTATTCTGACCAGACCGCTTCTAGAAAGTGAACTCGTAATC
GGAGCTGTGATCCTTCGTGGACATCTTCGTATTGCTGGACACCATCTAGGACGCTGTGACATCAAGGACC
TGCCTAAAGAAATCACTGTTGCTACATCACGAACGCTTTCTTATTACAAATTGGGAGCTTCGCAGCGTGT
AGCAGGTGACTCAGGTTTTGCTGCATACAGTCGCTACAGGATTGGCAACTATAAATTAAACACAGACCAT
TCCAGTAGCAGTGACAATATTGCTTTGCTTGTACAGTAA
